# Combined Cognitive-Behavioural and Physiotherapeutic Therapy for Patients with Chronic Pelvic Pain Syndrome (COMBI-CPPS): study protocol for a controlled feasibility trial

**DOI:** 10.1186/s13063-017-2387-4

**Published:** 2018-01-09

**Authors:** Christian A. Brünahl, Susanne G. R. Klotz, Christoph Dybowski, Björn Riegel, Sonja Gregorzik, Dean A. Tripp, Gesche Ketels, Bernd Löwe

**Affiliations:** 1 Department of Psychosomatic Medicine and Psychotherapy, University Medical Center Hamburg-Eppendorf, Martinistraße 52, 20246 Hamburg, Germany; 2 Department of Psychosomatic Medicine and Psychotherapy, Schön Klinik Hamburg Eilbek, Dehnhaide 120, 22081 Hamburg, Germany; 3 Department of Physiotherapy, University Medical Center Hamburg-Eppendorf, Martinistraße 52, 20246 Hamburg, Germany; 4 Department of Psychology, Queen’s University, Kingston, Ontario K7L 3 N6 Canada; 5 Department of Anaesthesia, Queen’s University, Kingston, Ontario K7L 3 N6 Canada; 6 Department of Urology, Queen’s University, Kingston, Ontario K7L 3 N6 Canada

**Keywords:** Chronic pelvic pain syndrome, Chronic pain, Cognitive behavioural therapy, Group psychotherapy, Physical therapy modalities, Feasibility studies

## Abstract

**Background:**

Chronic pelvic pain syndrome (CPPS) is a pain condition perceived in the pelvic area for at least 6 months. While evidence of the aetiology and maintenance of CPPS is still unclear and therapy options are rare, there is preliminary evidence for the efficacy of cognitive behavioural therapy and physiotherapy. However, an integrated treatment has not yet been studied. The primary aim of this study is therefore to test the feasibility of combined psychotherapy and physiotherapy for female and male patients with CPPS. The secondary aim is to explore changes in patient-relevant and economic outcomes compared to a control group.

**Methods:**

A feasibility study with a crossover design based on the principles of a ‘cohort multiple randomized controlled trial’ will be conducted to test a combined therapy for patients with CPPS. The study will consist of two consecutive treatment modules (cognitive behavioural group psychotherapy and physiotherapy as individual and group sessions), which will be applied in varying order. The modules will consist of nine weekly sessions with a 4-week break between the modules. The control group will undergo treatment as usual. Study subjects will be recruited from the interdisciplinary outpatient clinic for CPPS at the University Medical Center Hamburg-Eppendorf. Thirty-six patients will be assigned to the intervention, and 18 patients will be assigned to the control group. The treatment groups will be gender homogeneous. Feasibility as the primary outcome will be analysed in terms of the demand, acceptability, and practicality. Secondary study outcomes will be measured using validated self-rating-scales and physical examinations.

**Discussion:**

To the best of our knowledge, this study is the first to investigate the feasibility of combined psychotherapy and physiotherapy for patients with CPPS. In addition to testing feasibility, the results can be used for the preliminary estimation of therapeutic effects. The results from this study will be used to generate an enhanced therapeutic approach, which might be subject to further testing in a larger study.

**Trial registration:**

German Clinical Trials Register, DRKS00009976. Registered on 15 March 2016.

ISRCTN, ISRCTN43221600. Registered on 10 May 2016.

**Electronic supplementary material:**

The online version of this article (10.1186/s13063-017-2387-4) contains supplementary material, which is available to authorized users.

## Background

Chronic pelvic pain syndrome (CPPS) can be described as an intermittent or constant pain condition in the pelvic area that has persisted for at least 6 months without an obvious pathology that accounts for the pain [1]. It is associated with physical symptoms suggestive of gastroenterological, urogenital, and/or sexual dysfunction [1–3] as well as with psychopathological symptoms and a reduced health-related quality of life [1, 4–15]. Psychological correlates are also emphasized by clinical phenotyping systems, such as UPOINT [16]. Thirty-four to 37% of the patients with CPPS have positive findings in the UPOINT domain ‘psychosocial dysfunction’. Furthermore, 53–64% of the patients have findings in the ‘tenderness of muscles’ domain [17, 18], suggesting that psychotherapy and physiotherapy might be important in the treatment of patients with CPPS.

CPPS is a common pain condition with international general population prevalence rates ranging between 4 and 25% in women [8, 19–21] and between 2 and 18% in men [22–24].

Although CPPS is common, the aetiology and maintenance of CPPS are still largely unknown [25–29] and the successful management of this pain syndrome remains challenging [30, 31]. Several single-track medical and non-medical treatment strategies have failed to be sufficient [31, 32]. Therefore, a multidisciplinary approach combining medical, psychotherapeutic, and physiotherapeutic treatment strategies is recommended [1, 18, 33]. However, some psychotherapeutic and physiotherapeutic treatment strategies have shown promising effects. Cognitive behavioural therapy (CBT) strategies seem to reduce pain and symptom severity as well as increase the quality of life [34–36]. Myofascial physiotherapy techniques alone or in combination with breathing and relaxation techniques appear to be effective for treating urinary and sexual symptoms, pain, and quality of life [37–41].

### Objectives

Regarding the advocacy for multimodal therapy established in the guidelines of the European Association of Urology (EAU) [1], there is an urgent need to examine combined interventions for patients with CPPS. However, due to constraints of resources, not all interventions can be tested for efficacy and effectiveness. In this case, a feasibility study can be used to decide whether a treatment method is worth further investigation and whether changes should be applied to the intervention [42].

Therefore, the primary aim of this study is to explore the feasibility of a combined psychotherapeutic and physiotherapeutic treatment for both female and male patients with CPPS. The results from this study will be used to generate an enhanced therapeutic approach, which might be subject to further testing. Additionally, the secondary objective of this study is to determine the preliminary indicators for the efficacy of this treatment programme regarding urological symptoms, psychological and physical correlates, health-related quality of life, and healthcare utilization. The results can be used to calculate the optimal sample size for a randomized controlled trial (RCT).

## Methods/design

### Study design

This study will be conducted based on the principles of a ‘cohort multiple randomized controlled trial’ (cmRCT) proposed by Relton et al. [43]. In this pragmatic study design, an observational cohort of subjects with the parameter of interest will be recruited and evaluated on a regular basis. For a randomized controlled trial, random subjects from all eligible subjects in the cohort are allocated to the intervention group, while allocation to the control group is not randomized [43].

The feasibility study is embedded in the Interdisciplinary Research Platform Chronic Pelvic Pain Syndrome (CPPS), which was initiated in 2012 at the University Medical Center Hamburg-Eppendorf to obtain insight into the somatic and psychological aspects in CPPS and to develop treatment strategies for these patients. In cooperation with different medical specialties (e.g. psychosomatic medicine, urology, gynaecology, and physiotherapy), a specialized outpatient clinic for patients with CPPS was implemented [5]. The assessment at this outpatient clinic includes a diagnosis of CPPS according to the EAU guidelines [1]. People diagnosed with CPPS constitute the observational cohort, from which subjects for this study will be recruited.

**Fig. 1:**
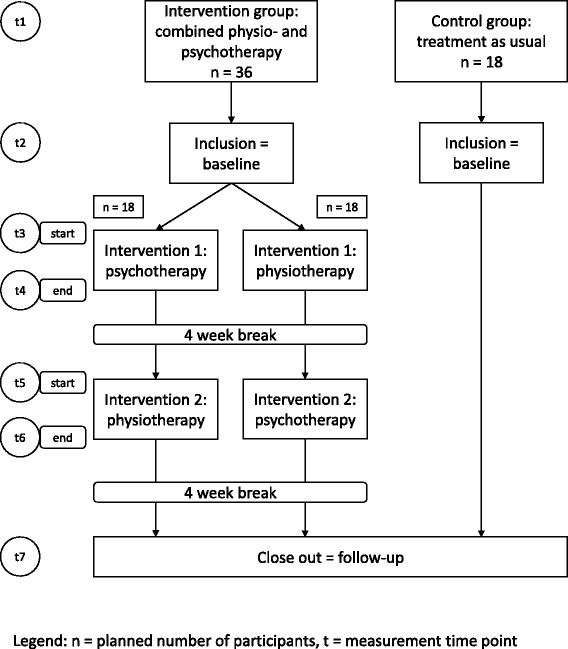
Overview of study procedure

The treatment will consist of a combination of cognitive behavioural psychotherapy and physiotherapy based on an aetiological model developed especially for patients with CPPS [6]. Psychotherapeutic and physiotherapeutic treatment modalities will be applied as consecutive modules, and both sequences will be tested (psychotherapy followed by physiotherapy vs physiotherapy followed by psychotherapy). The intervention will therefore consist of two branches, one starting with psychotherapy and the other starting with physiotherapy. For a detailed overview of the study design, see Fig. 1.

### Sample

Study subjects will be recruited from the observational cohort consisting of all patients assessed at the interdisciplinary outpatient clinic for CPPS at the University Medical Center Hamburg-Eppendorf.

The following criteria will be applied to identify eligible patients in the observational cohort: CPPS diagnosis according to the EAU guidelines [1] and classification of the International Association for the Study of Pain [44], informed consent, sufficient German language skills, age > 18 years, and score ≤ 40 for the mental or physical scale of the 12-Item Short-Form Health Survey (SF-12) [45]. Exclusion criteria are delusional disorders, substance dependence (except nicotine or pain medication), acute suicidal tendencies, planned absences over the treatment period, and current psychotherapy or physiotherapy.

The targeted sample size for the study is 54 participants. Thirty-six participants will be assigned to the intervention group and 18 to the control group. This sample size allows for evaluation of the study in terms of feasibility and can be used to estimate therapeutic effects (pre–post and between groups). Although the sample size is not sufficient to prove the efficacy of the combined treatment programme, the results of the study can be used to calculate the sample size for a subsequent RCT.

Assignment of eligible subjects to treatment and control groups will not be randomized; instead, it will be determined by the ability to regularly participate in the treatment sessions at the University Medical Center Hamburg-Eppendorf. Regular participation is defined as a maximum miss of four of the 18 treatment sessions. The assignment to one of the two treatment sequences (starting with psychotherapy vs starting with physiotherapy) will be randomized.

### Procedure

In a first step, all eligible patients who were examined in the interdisciplinary CPPS outpatient clinic since 2012 (time point t1), and are thus part of the observational cohort, will be identified and assigned to either the treatment group or the control group. Detailed information about the pilot study will be sent to these patients by postal mail, whereby the informed consent signed previously by patients for the assessment at the outpatient clinic facilitates contacting them for future research. Patients willing to participate in either the treatment group or the control group will undergo a telephone interview to re-examine eligibility in case changes have occurred since their visit to the outpatient clinic and to answer open questions about the study. After inclusion, participants will receive two copies of the informed consent document, the final time schedule and a set of questionnaires (time point t2; see Instruments for a detailed description). Participants of the treatment group will also be contacted by a physiotherapist to schedule an examination appointment. Patients who do not meet inclusion criteria will be informed by telephone and will receive support regarding alternative treatment options, if requested. Patients’ reasons for non-participation, if given, will be documented. In addition, patients who do not respond to the initial letter will also be contacted by telephone.

**Fig. 2:**
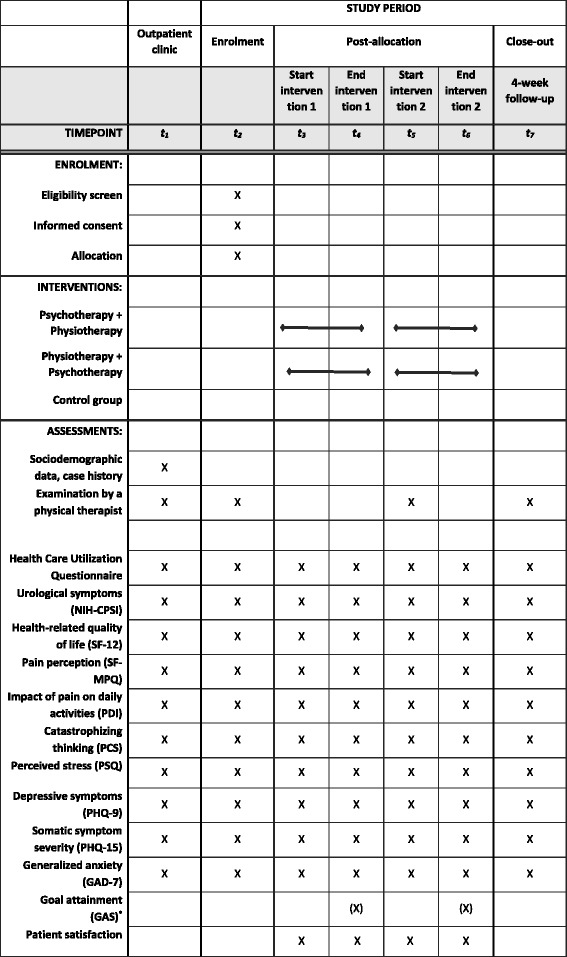
Standard Protocol Items: Recommendations for Interventional Trials (SPIRIT) schedule of enrolment, interventions, and assessments [46]. Legend: *GAD* = Generalized Anxiety Disorder Scale; *GAS* = Goal Attainment Scaling; *NIH-CPSI* = Chronic Prostatitis Symptom Index of the National Institute of Health; *PCS* = Pain Catastrophizing Scale; *PDI* = Pain Disability Index; *PHQ* = Patient Health Questionnaire; *PSQ* = Perceived Stress Questionnaire; *SF-MPQ* = Short-Form McGill Pain Questionnaire; *SF-12* = 12-Item Short-Form Health Survey; t = time point; * = only after the physical therapy intervention module (either at t4 or at t6)

Further measurements will be conducted at the beginning (t3) and end of the first intervention module (t4) and at the beginning (t5) and the end of the second intervention module (t6) as well as 4 weeks after finishing the second intervention module (t7). The study procedure is in line with the Standard Protocol Items: Recommendations for Interventional Trials (SPIRIT) statement 2013 [46] (see also Additional file 1: SPIRIT checklist). Figure 2 displays the schedule of enrolment, interventions, and assessments according to the SPIRIT statement.

### Intervention group

The intervention will consist of two consecutive treatment modules (cognitive behavioural group psychotherapy and physiotherapy as both group and individual sessions). A 4-week break is scheduled between the two modules. The intervention group has two branches; therefore, subjects will start with either one of the modules described in the following. A group size of nine patients for the psychotherapy as well as for the physiotherapy group sessions is regarded as adequate even in the event of drop-outs. This group size also reflects the maximal number of patients allowed in a CBT group in the German healthcare system [47]. The groups will be gender homogeneous because CPPS is characterized by symptoms in an intimate body region potentially associated with shame [48]. With a targeted sample size of 36 participants in the intervention and a group size of nine in the therapeutic sessions, the overall intervention group will consist of four therapeutic groups, two with only male participants and two with only female participants. One group of each gender will start with either psychotherapy or physiotherapy, resulting in four treatment groups in the intervention group.

### Cognitive behavioural psychotherapy

The psychotherapeutic intervention will consist of nine weekly group sessions, each lasting 90 minutes. The sessions will be based on the following pattern: group discussion of assignments (behaviour analysis, reading a particular chapter from the patient workbook described in the following), progressive muscle relaxation (PMR) according to Jacobson [49], session-specific theory, consolidation of the specific theory through group work, concluding round, and new assignments. For a detailed overview of the CBT, see Table 1. Each session will be held by a trained and skilled CBT therapist (licensed psychotherapist) and a co-therapist (resident physician); one will be male and the other female. In order to increase generalizability we have a pool of five therapists (three female, two male) who can deliver the study intervention. All therapists will receive in-house training especially for the study and will be supervised by one specialist in CBT. During the initial session, patients will receive a printed version of the patient workbook containing theoretical background information, assignments, and repeated questionnaires regarding their symptoms for the self-evaluation of their course.

**Table 1:** Overview of cognitive behavioural group psychotherapy sessions

Session	Content	Modality
1	Introduction to the programme; issuing of the patient workbook; overview of key topics; introduction to PMR	Group (90 min)
2	Group discussion/debriefing of Chapter 1 of the patient workbook; exercise of PMR; behaviour analysis	Group (90 min)
3	Group discussion/debriefing of Chapter 2 of the patient workbook; exercise of PMR; theory: catastrophizing cognitions; behaviour analysis	Group (90 min)
4	Group discussion/debriefing of Chapter 3 of the patient workbook; exercise of PMR; theory: negative self-talk; behaviour analysis	Group (90 min)
5	Group discussion/debriefing of Chapter 4 of the patient workbook; exercise of PMR; theory: influence of social relationships (Part 1); modification of ‘I-message’; behaviour analysis (focus: social interaction)	Group (90 min)
6	Group discussion/debriefing of Chapter 5 of the patient workbook; exercise of PMR; theory: influence of social relationships (Part 2)/asking for support; modification of listening skills; behaviour analysis	Group (90 min)
7	Group discussion/debriefing of Chapter 6 of the patient workbook; exercise of PMR; theory: coping strategies (Part 1)/role of positive self-messages; behaviour analysis	Group (90 min)
8	Group discussion/debriefing of Chapter 7 of the patient workbook; exercise of PMR; theory: coping strategies (Part 2); activity and inactivity/recognizing avoidance behaviour; behaviour analysis	Group (90 min)
9	Group discussion/debriefing of Chapter 8 of the patient workbook; exercise of PMR; assessment of changes during the programme; revision of key topics	Group (90 min)

*min* minutes, *PMR* progressive muscle relaxation

The patient workbook for cognitive behavioural group psychotherapy has been designed by members of our study group, and is based on the work of Tripp, Nickel, and Mullins [50, 51] who developed a treatment rationale for individual therapy and demonstrated its feasibility and yielded first indicators of its efficacy [35]. Through cooperation with the Canadian workgroup, we were able to translate, expand, and adapt their patient workbook [51] to the needs of our study and the German healthcare system. Key topics for the cognitive behavioural intervention are as follows:

- coping with catastrophizing cognitions,
- reduction of avoidance behaviour/increase of physical activity,
- development of coping strategies, and
- enhancing social support.

Furthermore, behaviour analysis also plays a key role in the programme. As group therapy facilitates the acquisition of new behaviour patterns [52], behaviour changes are addressed in the group setting. To increase the possibility of implementation into the German healthcare system we adapted the workbook to a group context.

### Physiotherapy

Following the structure of the psychotherapeutic intervention, the physiotherapeutic approach is also designed in nine weekly units. However, unlike the sessions in the psychotherapy, only units 1, 5, and 9 are group treatments, while the others are designed as individual appointments. The group sessions will last 90 minutes each, and the individual sessions will last 60 minutes except for the seventh unit, which will last 90 minutes and include treatment as well as feedback and reflection about the achievement of patients’ goals. Because of the more intense activity during the individual treatment and framework of ambulatory physiotherapy in the German healthcare system [53], a shorter duration was chosen in the single sessions.

The treatment is based on the Wise–Anderson Protocol, an American physiotherapeutic intervention for patients with CPPS combining trigger point therapy, a specific breathing technique, relaxation, and self-management [41, 54]. A German concept that acknowledges most of the elements of the American Wise–Anderson Protocol is Reflektorische Atemtherapie® [55, 56]. The German name of the concept is a registered trademark, and the English translation ‘reflective respiratory physiotherapy’ is from Zalpour [57]. This therapy aims to regulate psycho-physical coherences using the respiratory system. Specific stimuli of the connective tissue, muscles and tendons, joints, and periosteum are intended to influence the involuntary breathing and diaphragm activity. Hence, the aim is not only to improve the regulation of muscle tone and mobility, but also to affect the internal organs and pelvic floor through enhanced diaphragm mobility [58]. Positive effects of reflective respiratory physiotherapy were found in a study with patients who had chronic obstructive pulmonary disease [59].

The programme will contain the following elements [58, 60]:

- Education about the anatomy and function of the musculoskeletal system and posture with an emphasis on the pelvic floor and diaphragm, the influence of stress on the muscle tone and stiffness of fasciae, and the importance of self-management and adherence to a home exercise programme.
- Application of heat in the form of ‘hot towels’ (hot water-soaked towels) at the beginning of the therapy to relax muscles and joints, stimulate the circulation, and prepare the tissue for the following techniques.
- Manual techniques for all structures of the musculoskeletal system to mobilize joints and release fasciae with stretching and relaxing muscles.
- Specific therapeutic movements with partially uncomfortable or painful stimuli that influence the respiratory system and the diaphragm reflectively, affecting the vegetative nervous system and muscle tone.
- Instruction of the patient to self-management and home exercises based on yoga to strengthen and stretch muscles, improve posture and body perception, and sense breathing activity.

In the individual sessions, subjects will be treated according to their individual findings with ‘hot towels’, manual techniques, and specific therapeutic movements. In addition, home exercises will be taught. During the group sessions, the focus will be on home exercises and self-management together with education and information. Similar to the psychotherapeutic group sessions, the physiotherapy group sessions will be hosted by two physiotherapists, one male and one female. Table 2 presents a scheme for the procedure and content of the physiotherapeutic intervention.

**Table 2:** Overview of physiotherapy sessions

Session	Content	Modality
1	Relationship between muscle tension, stress, and pain; awareness of tension and relaxation of the pelvic floor muscles; instruction of home exercises/self-management; goal attainment scaling	Group (90 min)
2	Reflective respiratory physiotherapy; home exercises; awareness of changes during/after session	Single (60 min)
3	Reflection of the past sessions; reflective respiratory physiotherapy; home exercises; awareness of changes during/after session	Single (60 min)
4	Reflection of the past individual sessions; reflective respiratory physiotherapy; home exercises; awareness of changes during/after session	Single (60 min)
5	Reflection of the past group session; instruction of home exercises/self-management	Group (90 min)
6	Reflection of the past individual sessions; reflective respiratory physiotherapy; home exercises; working with the pain; awareness of changes during/after session	Single (60 min)
7	Reflection of the past individual sessions; reflective respiratory physiotherapy; home exercises; working with the pain; awareness of changes during/after session	Single (60 min)
	Feedback for the individual sessions; evaluation of and reflection on goal attainment; self-management	Single (30 min)
8	Reflection of the past individual sessions; reflective respiratory physiotherapy; home exercises; working with the pain; awareness of changes during/after session	Single (60 min)
9	Evaluation of and reflection on goal attainment; self-management; home exercises; feedback and conclusion	Group (90 min)

*min* minutes

### Control group

Allocation to the control group will not be randomized; instead, this will be determined by the ability to participate in the intervention occurring at the University Medical Center Hamburg-Eppendorf. It was considered difficult for patients outside the greater Hamburg area to participate; therefore, they will be allocated to the control group. The control group will not receive any specific intervention as part of the study; nonetheless, patients can seek treatment as usual from their local healthcare provider. Assessment of the control group will be done at two time points; first, at time point t2, which is the enrolment time; and second, at time point t7, which is 4 weeks after the intervention group has finished the second intervention module. The results of these measurements will be compared with the results of the intervention group to gather initial insight into the efficacy of the intervention compared to treatment as usual.

### Instruments

The assessment at our interdisciplinary CPPS outpatient clinic constitutes the measurement time point t1. This involves collection of socio-demographic data and the case history, an examination by a physiotherapist, and completion of psychometric questionnaires used in this study. For an overview of the instruments used in this study, see Fig. 2.

Feasibility will be operationalized using information from the participants, therapists, and those involved in organization of the study. Information from participants will include the response rate to study invitation, willingness to participate, and reasons for not participating as indicators of demand. Practicality will be operationalized in terms of the time and personnel expenditures. Attendance at and satisfaction with physiotherapy and psychotherapy sessions, the number of drop-outs and adverse events, and the amount of missing data in the questionnaires of the workbook will function as indicators of acceptability. To assess satisfaction, we developed questionnaires using 7-point Likert scales. Subjects will be asked to rate each psychotherapeutic and physiotherapeutic session, including the accompanying study materials, each whole treatment module (psychotherapy or physiotherapy), and overall contentment with the combination of psychotherapy and physiotherapy. The questionnaires cover therapeutic and organizational aspects.

The secondary objectives of the feasibility study will be measured using the following instruments:

- The health-related quality of life will be assessed using the SF-12 [45], which has been demonstrated as reliable and valid in clinical and population-based samples [61, 62].
- The Chronic Prostatitis Symptom Index of the National Institute of Health (NIH-CPSI) [63] is considered the criterion standard for assessing urological symptom severity in CPPS in the EAU guidelines [1]. The German version with good psychometric properties [64] will be applied in this study. Since the original NIH-CPSI was designed for male patients, a modified version for female patients also exists [65].
- The German version [66] of the Short-Form McGill Pain Questionnaire (SF-MPQ) [67] will be used to assess pain perception.
- The impact of pain on the ability to participate in essential life activities will be measured with the Pain Disability Index (PDI) [68, 69], a valid and reliable [70] instrument.
- Pain catastrophization will be assessed with the aid of the Pain Catastrophizing Scale (PCS) [71], which has been shown to have good psychometric properties [72].
- To quantify the psychological symptom burden, three subscales of the German version of the Patient Health Questionnaire (PHQ-D) [73] with good psychometric characteristics [74–76] will be applied: the PHQ-9 for measuring depressive symptoms [77], the PHQ-15 for measuring the severity of somatic symptoms [78], and the Generalized Anxiety Disorder Scale (GAD-7) [76, 79] for measuring symptoms of generalized anxiety.
- The reliable and valid [80] German short version [81] of the Perceived Stress Questionnaire (PSQ) [82] will be used to assess subjectively experienced stress.
- Assessment of tender and trigger points in the abdominal wall, bottom, thighs, and pelvic floor is done with external and internal manual palpation. Although the reliability of manual palpation is variable [83, 84], it is essential in finding painful points in the muscles [85–87]. In female subjects, internal palpation is done via the vagina and rectum; in male subjects, internal palpation is done via the rectum. Prior to this examination, patients gave written informed consent to internal palpation.
- Participants set their individual therapy goals on the participation level of the International Classification of Functioning, Disability and Health [88] in the first physiotherapeutic group session and evaluate them in the last group treatment using the reliable and valid [89–92] Goal Attainment Scaling (GAS) [93].
- To assess healthcare utilization, we are using the Health Care Utilization Questionnaire, which is a modified version of the Client Socio-Demographic and Service Receipt Inventory—European Version [94] and was developed by the Institute of Health Economics and Health Services Research of the University Medical Center Hamburg-Eppendorf.

### Data management and analysis

After completion of data collection, raw data will be entered in prepared electronic databases and merged with the electronically captured data. The accuracy of data will be checked by two independent researchers. Data saving and storage will be performed in accordance with the German regulation of Good Clinical Practice [95].

In addition to the quantitative data, feasibility will be analysed using qualitative data, such as answers to open questions in the satisfaction questionnaires and verbal information.

Descriptive statistics will be used to summarize the sample characteristics (e.g. sex, age, and symptom duration) and two-tailed independent *t-*tests will be used to test for significant differences between the intervention and control groups at enrolment (t2).

Subjects will be analysed on an intention-to-treat basis. To examine the course of the symptoms, related variables will be analysed using the pre–post point estimate comparisons, variability estimates, and 95% confidence intervals. The controlled study design allows for within-group as well as between-group comparisons. Paired-sample *t*-tests will be used for within-group comparisons, while the independent *t-*test will be used for between-group comparisons.

The significance level for all *t-*tests will be set at *p* < 0.05.

The analyses of the course of the symptom-related variables will function as estimates of the effect sizes, while effect estimates can be obtained for physiotherapy and psychotherapy separately as well as the overall effect estimates. These estimates can be used to determine the optimal sample size for a subsequent RCT with a normally distributed sample; hence, parametric tests will be applied as statistical procedures in the feasibility study. Factors influencing therapy success will also be examined.

Statistical analyses will be performed with IBM SPSS Statistics, Version 24 (IBM, Armonk, NY, USA).

## Discussion

This article describes the research protocol for a controlled feasibility study of a combination of psychotherapeutic and physiotherapeutic treatments for patients with CPPS. The study will use an interdisciplinary short-term group intervention consisting of psychotherapy and physiotherapy for testing feasibility of the combined intervention as well as providing the first indicators of efficacy.

The group assignment will be based on the ability of regular participation in the intervention which might lead to selection bias. However, we deemed regular attendance important for the positive effect of the whole intervention programme, and as the complete intervention will last 22 weeks (each intervention module has a duration of 9 weeks with a 4-week break in between) it will require a great concession in terms of time. Participants will not only have a weekly appointment at University Medical Center Hamburg-Eppendorf, they will also have to prepare the psychotherapeutic sessions by reading the workbook chapters and completing the respective questionnaires. It is unclear whether patients will comply with these requirements so that they will be prepared enough to follow and understand the content of the single psychotherapeutic sessions. Moreover, it is expected that at least some subjects will miss one or more sessions due to shift work, unplanned vacations, or other reasons. This might result in difficulties in understanding the content of the subsequent sessions, influencing the effect of the intervention. However, the subjects will have manuals for both the psychotherapy and physiotherapy components, which will allow them to educate themselves even if they have missed a session. Both intervention modules will be applied in a subsequent order rather than to deliver physiotherapy and psychotherapy at the same time. This approach was chosen so that participants have to make time for a weekly appointment and estimate the effects of each module separately. Nonetheless, some patients might find it tempting to select the intervention module they find more interesting or suitable for their individual situation and skip the other one. In addition, the subsequent order contributes to the prolongation of the overall treatment period. All psychotherapy sessions will be provided as group treatments. Group sessions will be accompanied by a workbook, which requires that participants adhere to specific assignments and may influence their motivation. Nonetheless, the workbook provides support and advice both during the intervention period and after its completion.

Prior studies suggest that physiotherapy is highly valued by patients with CPPS [6, 96] and can empower them to take responsibility for themselves and their coping with pain [97]. During the design of the intervention, the aspect of empowerment and self-management was emphasized, which was a strength of the study. Moreover, instead of adapting a foreign concept such as the Wise–Anderson Protocol [54], a German, already implemented, physiotherapeutic management approach was used. The combination of physiotherapeutic group and individual sessions is not part of the regular health care in ambulatory settings in Germany and might be unexpected for some participants. While they will be in a confidential setting during individual treatments with the physiotherapist, they will have to cope with several other patients being present during performance of exercises. Nevertheless, this group experience can also have a positive effect on the subjects.

We intend to recruit patients from the CPPS outpatient clinic, which has been ongoing since 2012 and serves as the observational cohort in our study design. This cohort is limited in size, and it could be brought into question whether sufficient patients are willing to participate and fulfil eligibility criteria. Their initial assessment at the outpatient clinic might be several months to years prior and their situation with regard, but non-exclusive, to the CPPS might have changed, resulting in non-participation in the study. However, this feasibility study should provide information for further optimization of the treatment approach and power calculation in future RCTs rather than sufficient testing of programme effects. Because of the exploratory nature of the study, no sample calculation was performed, and the selection of controls was based on pragmatic reasons. Nevertheless, to the authors’ knowledge, this study is the first to evaluate a combined programme of psychotherapy and physiotherapy for patients with CPPS while acknowledging the multifactorial aetiology and demand for multimodal therapies [1, 17].

### Trial status

The study is currently ongoing. Recruitment of patients started in mid-May 2016 and will continue until the targeted sample size is reached. The first two groups, one that started with physiotherapy and the other with psychotherapy, underwent treatment from June to November 2016. The second two groups started in January 2017 and will be treated until June 2017. The next two groups are supposed to start treatment in July 2017.

## Additional file

Additional file 1: Standard Protocol Items: Recommendations for Interventional Trials (SPIRIT) checklist (DOC 120 kb)

## Abbreviations

CBT: Cognitive behavioural therapy
cmRCT: Cohort multiple randomized controlled trial
CPPS: Chronic Pelvic Pain Syndrome
DSM-IV: Diagnostic and Statistical Manual of Mental Disorders IV
EAU: European Association of Urology
GAD-7: Generalized Anxiety Disorder Scale
GAS: Goal Attainment Scaling
NIH-CPSI: Chronic Prostatitis Symptom Index of the National Institute of Health
PCS: Pain Catastrophizing Scale
PDI: Pain Disability Index
PHQ: Patient Health Questionnaire
PMR: Progressive muscle relaxation
PSQ: Perceived Stress Questionnaire
RCT: Randomized controlled trial
SCID: Structured Clinical Interview for DSM-IV Axis I Disorders
SF-12: 12-Item Short-Form Health Survey
SF-MPQ: Short-Form McGill Pain Questionnaire
